# Rapidly Declining SARS-CoV-2 Antibody Titers within 4 Months after BNT162b2 Vaccination

**DOI:** 10.3390/vaccines9101145

**Published:** 2021-10-08

**Authors:** Dong-Ho Jo, Dohsik Minn, Jaegyun Lim, Ki-Deok Lee, Yu-Min Kang, Kang-Won Choe, Kwang-Nam Kim

**Affiliations:** 1 Division of Infectious Disease, Department of Internal Medicine, Myongji Hospital, Goyang 10475, Korea; feeldhy@hanmail.net (D.-H.J.); kdlee.snu.eulji@gmail.com (K.-D.L.); notjustblue@gmail.com (Y.-M.K.); choekw@mjh.or.kr (K.-W.C.); 2 Seegene Medical Foundation, Seoul 05548, Korea; dsmin@mf.seegene.com; 3 Department of Laboratory Medicine, Myongji Hospital, Goyang 10475, Korea; jaegyun@mjh.or.kr; 4 Department of Pediatrics, Myongji Hospital, Goyang 10475, Korea

**Keywords:** SARS-CoV-2, vaccine immunogenicity, BNT162 vaccine, antibodies, neutralizing antibodies

## Abstract

The efficacy and safety of the BNT162b2 vaccine are known, but antibodies are expected to decrease over time after vaccination. We collected blood samples from 104 fully vaccinated health care workers at 3 and 5 weeks after first vaccination and 4 months after second vaccination. Antibody titers and neutralizing antibodies were measured. In our study, both antibody titers and neutralizing antibodies increased significantly at 5 weeks after first vaccination but decreased rapidly at 4 months after second vaccination. Additionally, the results showed a significant decrease regardless of gender or age. Further studies are needed to help determine the interval of SARS-CoV-2 vaccinations.

## 1. Introduction

According to the WHO, as of 1 October 2021, 233,503,524 people were infected with COVID-19 worldwide, of whom 4,777,503 died [1]. The purpose of vaccination is to prevent infectious diseases by activating humoral and cellular immunity. Vaccines are currently being researched and developed using various platforms, which range from traditional to new methods [2]. Over 202 candidates for the severe acute respiratory syndrome coronavirus 2 (SARS-CoV-2) vaccine are currently being studied using various methods [1]. The BNT162b2 vaccine is a novel type of vaccine that uses mRNA [3]. The effectiveness and safety of the BNT162b2 vaccine have been proven through previous studies, including clinical trials [3,4]. Several vaccines have gained emergency use authorization less than a year after the emergence of SARS-CoV-2, which is an exceptional event in vaccine history. However, since SARS-CoV-2 vaccines such as BNT162b2 have a short history of development, the level of antibody persistence and immunity against infectious diseases remain unclear. 

Data from the Korea Centers for Disease and Prevention indicate that 7772 fully vaccinated individuals (0.044% of the 17,752,946 vaccinations) had experienced breakthrough infections as of 19 September 2021 [5]. At the same time, 3 of 1779 fully vaccinated health care workers (HCWs) were diagnosed with breakthrough infections at our center. Breakthrough infections with SARS-CoV-2 are reportedly associated with low neutralizing antibody titers at the time of diagnosis [6]. However, we do not yet clearly know the correlation between antibody titers and breakthrough infections because SARS-CoV-2 vaccinations were started in 2021. Antibody titers also decreased over time following the administration of other vaccinations, and others require regular booster vaccinations, such as influenza vaccines [7]. Therefore, knowing the degree of decrease in antibody titers over time and looking at the trend of breakthrough infections will help determine the vaccination interval. Additionally, it would be helpful to measure the level of neutralizing antibodies that are directly involved in the prevention of infectious diseases, especially among the total antibodies generated after vaccinations.

In the present study, we aimed to investigate the long-term immunity induced by SARS-CoV-2 vaccines. To the best of our knowledge, this is the first follow-up study on the durability of antibodies after BNT162b2 vaccination in Korea.

## 2. Materials and Methods

Our study was a prospective observational cohort study in which antibody and neutralizing antibody titers were continuously assessed after vaccination with the Pfizer (BNT162b2) vaccine, which is mRNA-based. The vaccinations were administered on March 12 (first vaccination) and 2 April 2021 (second vaccination) at a 3-week interval. Among the 208 fully vaccinated HCWs at Myongji Hospital, 104 consented to participate in the study and were enrolled.

Blood samples were collected at 3 and 5 weeks after the first vaccination and 4 months after the second vaccination. The number of anti-S antibodies (including IgG) to the SARS-CoV-2 spike protein receptor-binding domain (RBD) was measured, while the neutralizing antibodies were assessed based on their signal inhibition rate (SIR, %, cut-off value 30%) with a Genscript kit (ELISA-based SARS-CoV-2 Surrogate Virus Neutralization Test Kit). This kit shows results similar to those of the plaque reduction neutralization test, which is considered the gold standard for measuring antibody levels against various viral diseases [8]. SIR was used to assess the capacity of antibodies to neutralize, in this case, a surrogate virus. The cPass™ ELISA-based SARS-CoV-2 surrogate virus neutralization antibody detection test kit (GenScript Biotech Corporation, Piscataway, NJ, USA), applies an indirect detection method via assessing antibody-mediated inhibition of SARS-CoV-2 RBD binding to the human host receptor angiotensin-converting enzyme 2. The percentage inhibition was calculated using the equation below. For interpretation of the data, we applied a 30% cut-off value; samples were deemed positive if they exhibited an SIR greater than 30% and negative if the rate was less than 30%.

$$\mathrm{Inhibition}=(1-\frac{\mathrm{Optical}\;\mathrm{density}\;\mathrm{value}\;\mathrm{of}\;\mathrm{sample}}{\mathrm{Optical}\;\mathrm{density}\;\mathrm{of}\;\mathrm{negative}\;\mathrm{control}})\times 100$$

Differences between continuous variables in the vaccination groups were evaluated using the t-test. The data were analyzed using R software (version 4.0.3; R Foundation for Statistical Computing, Vienna, Austria). Statistical significance was set at a 2-sided *p*-value < 0.05.

The institutional review board (IRB) of Myongji Hospital approved this study (IRB No. MJH-2021-07-053). Informed consent was obtained from all participants enrolled in the study.

## 3. Results

### 3.1. Demographics Data

The number of participants included in the present study was 104 at 3 and 5 weeks after the first vaccination and 83 at 4 months after the second vaccination. A total of 59 (56.7%) women participated at 3 and 5 weeks after first vaccination and 48 (57.8%) at 4 months after second vaccination. The mean age at both 3 and 5 weeks was 43.3 years and 44 years at 4 months (Table 1).

### 3.2. Total Anti-S Antibody Titer and SIR

**Figure 1:**
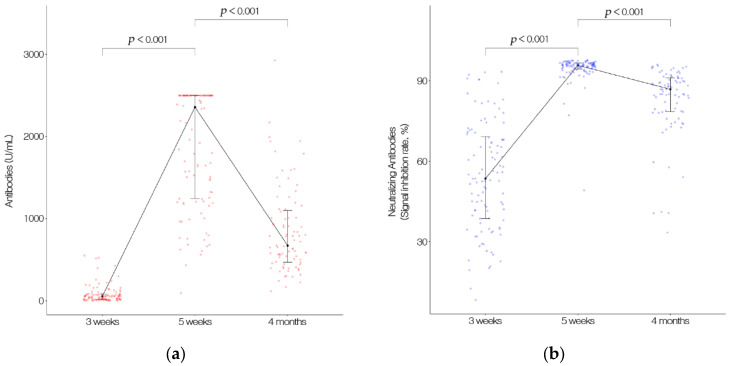
(**a**) The total anti-S antibodies (including IgG) to the SARS-CoV-2 spike protein receptor binding domain (RBD) counts. Data were measured at 3 and 5 weeks after first vaccination and 4 months after second vaccination. (**b**) Neutralizing antibodies counts. Data were measured at 3 and 5 weeks after first vaccination, and 4 months after second vaccination with an ELISA-based SARS-CoV-2 Surrogate Virus Neutralization Test Kit.

The total anti-S antibody counts were 82.4 U/mL, 1893.0 U/mL, and 851.7 U/mL at 3 weeks, 5 weeks, and 4 months, respectively. The antibody titer was significantly higher at 5 weeks than at 3 weeks after the first vaccination. It then decreased significantly 4 months after the second vaccination. The SIRs were 54.4%, 94.5%, and 82.8% at 3 weeks, 5 weeks, and 4 months, respectively and decreased significantly at 4 months after the second vaccination, similar to the antibody titer (Figure 1).

### 3.3. Difference in Antibody Titer and SIR According to Gender

**Figure 2:**
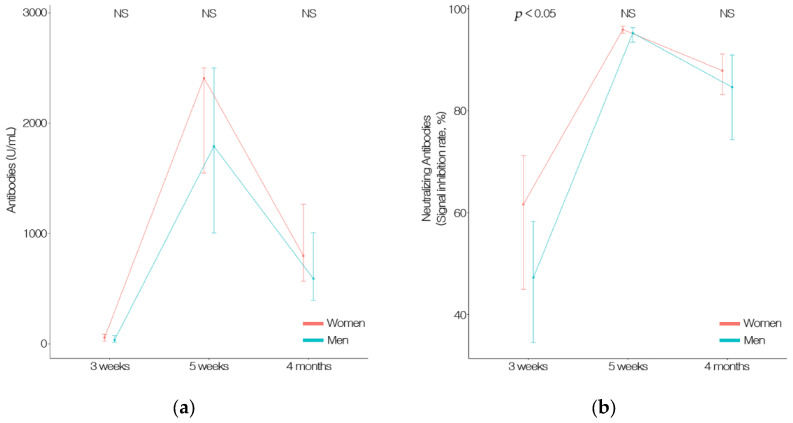
(**a**) The total anti-S antibodies (including IgG) to the SARS-CoV-2 spike protein receptor-binding domain (RBD) counts by gender. Data were measured at 3 and 5 weeks after first vaccination and 4 months after second vaccination. (**b**) Neutralizing antibodies counts by gender. Data were measured at 3 and 5 weeks after first vaccination, and 4 months after second vaccination with an ELISA-based SARS-CoV-2 Surrogate Virus Neutralization Test Kit.

Although the difference in antibody titer was higher in women than in men (3 weeks, 96.0 U/mL; 5 weeks, 2004.6 U/mL; 4 months, 929.3 U/mL; versus 3 weeks, 64.5 U/mL; 5 weeks, 1746.7 U/mL; 4 months, 745.3 U/mL), it was not significant. The SIR of women (58.8%) was significantly higher than that of men (48.7%) at 3 weeks, (*p* < 0.05); however, no significant difference was observed at 5 weeks (95.1% and 93.6% for women and men, respectively) or 4 months (85.3% and 79.4% for women and men, respectively). The SIR tended to be higher in women. However, both the antibody titer and SIR were significantly lower in both men and women at 4 months after the second vaccination (Figure 2 and Table 2).

### 3.4. Difference in Antibody Titer and SIR According to Age

**Figure 3:**
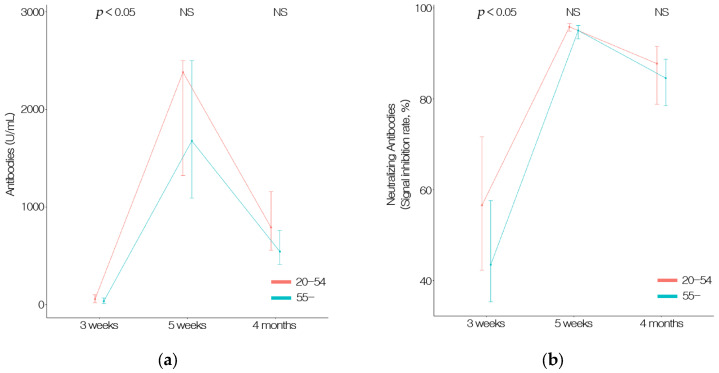
(**a**) The total anti-S antibodies (including IgG) to the SARS-CoV-2 spike protein receptor-binding domain (RBD) counts by age. Data were measured at 3 and 5 weeks after first vaccination and 4 months after second vaccination. (**b**) NeuTable 3 and 5 weeks after first vaccination and 4 months after second vaccination with an ELISA-based SARS-CoV-2 Surrogate Virus Neutralization Test Kit.

The antibody titer was 94.0 U/mL and 47.6 U/mL in the young (20 to 54 years old) and old-age groups (55 years or older) at 3 weeks, respectively (*p* < 0.05). No significant differences were observed between the young and old age groups at 5 weeks (1934.8 U/mL and 1767.6 U/mL, respectively) or 4 months (905.2 U/mL and 703.3 U/mL, respectively). Similarly, the young age group had a significantly higher SIR than the old age group at 3 weeks (57.2% and 46.1%, respectively). However, the difference in SIR between the young and old age groups at 5 weeks (95.0% and 92.9%, respectively) and 4 months (83.9% and 79.7%, respectively) was not significant. Overall, the younger age group tended to have higher SIRs. Changes due to age were also significantly higher at 5 weeks after the first vaccination but decreased significantly at 4 months after the second vaccination (Figure 3 and Table 3).

## 4. Discussion

In this study, we found that the antibody and neutralizing antibody titers increased significantly at 2 weeks after the second dose of the BNT162b2 vaccine and decreased sharply at 4 months. Overall, the antibody and neutralizing antibody titers tended to be higher among women and younger individuals, but the groups’ values did not differ significantly, and all subgroups exhibited a rapid reduction in these values 4 months after the second vaccination. 

Previous studies also demonstrated that a marked gradual decrease in the numbers of antibodies and neutralizing antibodies occurred after vaccination [9,10]. This was similar to other previously gathered data, which reported that antibody titers tended to be higher among women and younger individuals [9]. Although the measured units were different, similar to our study, antibody titers were the highest immediately after full vaccination [9,10]. Antibody levels also decrease over time even after natural SARS-CoV-2 infection [11,12]. Similarly, antibodies increased after vaccination and remained higher than those of non-infected groups, but a gradual decline also occurred after vaccination [10]. 

The level of antibody titer reduction and the resulting susceptibility to infection remains unknown. Another study found that 608 of 33,993 vaccinated patients had positive SARS-CoV-2 polymerase chain reaction (PCR) test results. The group vaccinated within 146 days had a significantly lower positive rate than the group tested 146 days or longer after vaccination [13]. Both studies indicated that antibodies decreased over time, resulting in reduced immunity to infections [6,13]. 

Although vaccines differ, a longitudinal study following the influenza pandemic vaccination revealed that antibodies decreased 12 months after vaccination. Annual influenza vaccination was recommended for HCWs based on the same study [7]. As a result, the SARS-CoV-2 vaccine also requires continuous follow-up research, and the vaccination interval should be determined based on when immunity decreases. The administration of booster vaccinations in HCWs should also be discussed since they are a high-risk group, as in influenza vaccines.

This study has several limitations. First, this was a single-center study; however, our study had the advantage of observing the same participants longitudinally. Second, the antibody levels did not differ according to age; however, no children or adolescents were included in the HCW population, and the older population was relatively small compared to the general population. According to one study, antibody titers may be relatively low in older people [9]. These differences may result in changes in vaccination strategies based on age; therefore, additional studies on age-specific antibody titers in children and elderly individuals are needed. Third, our study was relatively short, having only a 4-month follow-up. Fourth, cellular immunity was not tested in the present study. A reduction in antibody levels does not necessarily indicate a decrease in immunity. Our immune mechanisms are diverse, and memory B cells and cell-mediated immunity can provide long-term immunity along with antibodies. The number of antibodies to RBD decreased after 6 months; however, memory B cells continue to increase for 3–6 months, and antigen-specific CD8^+^ T cell and memory CD4^+^ T cell responses were maintained [14].

## 5. Conclusions

In conclusion, the numbers of antibodies and neutralizing antibodies rapidly decreased after vaccination. Additionally, there was no significant difference in this rapid decline according to gender or age. A long-term follow-up study is needed to determine the optimal interval between booster and regular vaccinations, and further mechanisms to attain immune responses from the new vaccines should be identified.

## Figures and Tables

**Table 1:** Demographics of participants.

	3 Weeks (N = 104)	5 Weeks (N = 104)	4 Months (N = 83)
Sex			
Men	45 (43.3%)	45 (43.3%)	35 (42.2%)
Women	59 (56.7%)	59 (56.7%)	48 (57.8%)
Age			
Mean (SD)	43.3 (15.5)	43.3 (15.5)	44.0 (15.8)
Median [Min, Max]	41.0 [24.0, 82.0]	41.0 [24.0, 82.0]	41.0 [24.0, 82.0]
Age group			
20–54	78 (75.5%)	78 (75.5%)	61 (73.5%)
55-	26 (24.5%)	26 (24.5%)	22 (26.5%)

**Table 2:** Antibody titer and neutralizing antibodies according to gender.

	Men	Women	*p*-Value
3 weeks			
Antibodies (U/mL)	64.5 ± 95.2	96.0 ± 118.0	NS
Neutralizing antibodies (SIR, %)	48.7 ± 19.0	58.8 ± 20.3	*p* < 0.05
5 weeks			
Antibodies (U/mL)	1746.7 ± 769.3	2004.6 ± 632.8	NS
Neutralizing antibodies (SIR, %)	93.6 ± 7.2	95.1 ± 3.4	NS
4 months			
Antibodies (U/mL)	745.3 ± 551.3	929.3 ± 503.2	NS
Neutralizing antibodies (SIR, %)	79.4 ± 15.7	85.3 ± 10.2	NS

**Table 3:** Antibody titer and neutralizing antibodies according to age.

	20–54	55–	*p*-Value
3 weeks			
Antibodies (U/mL)	94.0 ± 121.3	47.6 ± 47.4	*p* < 0.05
Neutralizing antibodies (SIR, %)	57.2 ± 20.3	46.1 ± 18.1	*p* < 0.05
5 weeks			
Antibodies (U/mL)	1934.8 ± 687.7	1767.6 ± 748.7	NS
Neutralizing antibodies (SIR, %)	95.0 ± 3.2	92.9 ± 9.2	NS
4 months			
Antibodies (U/mL)	905.2 ± 536.2	703.3 ± 488.5	NS
Neutralizing antibodies (SIR, %)	83.9 ± 11.8	79.7 ± 16.0	NS

## Data Availability

Data is contained within the article.
